# Developmental attenuation of N-methyl-D-aspartate receptor subunit expression by microRNAs

**DOI:** 10.1186/s13064-015-0047-5

**Published:** 2015-09-17

**Authors:** Caroline Corbel, Israel Hernandez, Bian Wu, Kenneth S. Kosik

**Affiliations:** Neuroscience Research Institute, Department of Molecular, Cellular and Developmental Biology, University of California, Santa Barbara, CA 93106 USA; Present address: EA4250-Laboratoire d’Ingénierie des Matériaux de Bretagne, Equipe Génie des Bioprocédés et Biomolécules, Université de Bretagne Sud, CER Yves Coppens, Vannes, 56017 France

**Keywords:** microRNA (miRNA), Synaptic plasticity, Glutamate receptor, Developmental switch, NMDA receptor, miR-19a, miR-539

## Abstract

**Background:**

N-methyl-D-aspartate receptors (NMDARs) are a subtype of ionotropic glutamate receptors and are expressed throughout the central nervous system (CNS). Their activity is required for excitatory synaptic transmission, the developmental refinement of neural circuits and for the expression of many forms of synaptic plasticity. NMDARs are obligate heterotetramers and the expression of their constituent subunits is developmentally and anatomically regulated. In rodent cortex and hippocampus, the GluN2B subunit is expressed at high levels early in development and decreases to plateau levels later while expression of the GluN2A subunit has a concomitant increase. Regulation of GluN2A and GluN2B expressions are incompletely understood. Here, we showed the influence of miRNAs in this process.

**Findings:**

Two miRNAs, miR-19a and miR-539 can influence the levels of NMDARs subunits, as they target the mRNAs encoding GluN2A and GluN2B respectively. MiR-539 also modified the expression of the transcription factor REST, a known regulator of NMDAR subunit expression.

**Conclusions:**

miR-19a and miR-539, in collaboration with REST, serve to set the levels of GluN2A and GluN2B precisely during development. These miRNAs offer an entry point for interventions that affect plasticity and a novel approach to treat neurodegenerative diseases.

## Findings

N-methyl-D-aspartate receptors (NMDARs) are a subtype of ionotropic glutamate receptors and are widely expressed throughout the nervous system [1]. They are required for induction and expression of many forms of synaptic plasticity [2] and are implicated in the developmental refinement of neural circuits [3]. NMDARs are obligate heterotetramers, requiring two GluN1 subunits and two other subunit types (GluN2A-D or GluN3A-B). In the cortex and hippocampus, GluN2B expression is initially high in neurons, but decreases during development as the expression of GluN2A increases [4]. These shifting expression patterns are thought to affect the threshold for and the magnitude of long-term potentiation (LTP) [5, 6]. The experimental elimination of GluN2B in the adult increased the number of functional synapses and the absence of GluN2A increased the strength of unitary connections [7, 8]. GluN2B subunit-containing NMDA receptors promote plasticity-induced spine growth [9] and hippocampal-dependent learning [10]. NMDARs mediate the synaptotoxic effects of β-amyloid oligomers on LTP [11–13] and excitotoxicity [14]. Although GluN2A and GluN2B subunits are clearly involved in many developmental and pathological processes, the molecular factors controlling subunit expression are incompletely understood. The only control element so far identified is the transcriptional repressor REST, which maintains the expression of GluN2B [15].

MicroRNAs (miRNAs) regulate gene expression post-transcriptionally and typically undergo large profile shifts in expression when cells change identity during development or oncogenesis [16, 17]. However, as the fate of a neuron narrows during development the role of miRNAs is less clear. A recent study implicated miR-124 in the distribution and regulation of another glutamate receptor subunit, GluA2 [18]; however, the involvement of miRNAs in the expression of NMDAR subunits is heretofore unreported. Here, we demonstrate that miR-19a and miR-539 expression patterns are complementary to those of GluN2A and GluN2B.

## Results

### MicroRNA control over developmental switching of NMDAR subunits

**Fig. 1:**
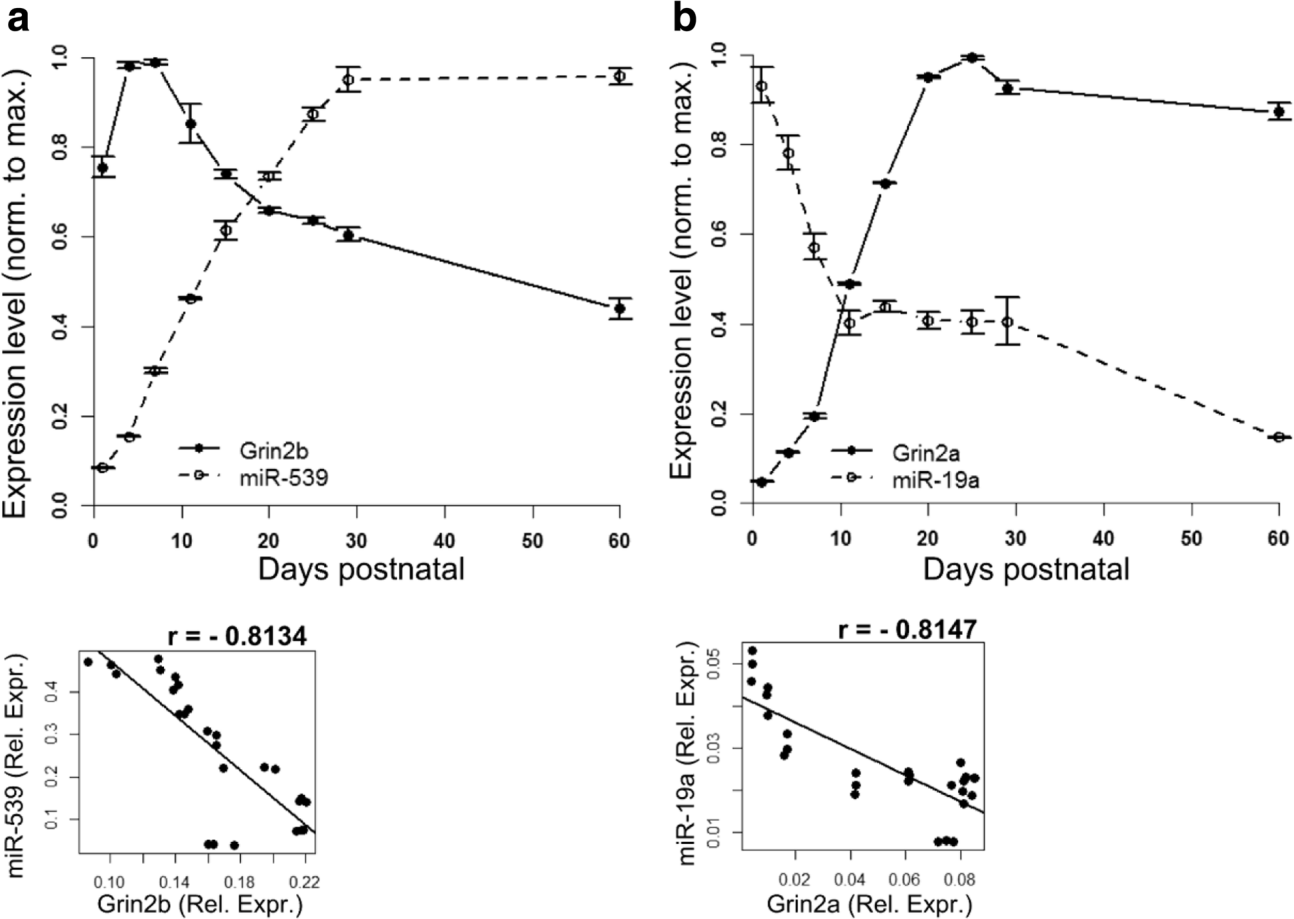
**a**, **b**. Expression of mRNAs encoding the NMDARs in rat hippocampi. qPCR analysis showing the relative expression levels of Grin2a and Grin2b mRNAs and their targeting miRNAs during development (from P1 to P60) in hippocampi (n = 3). The correlation coefficients are specified at the bottom of both graphs

The expression pattern of Grin2a and Grin2b (mRNAs for GluN2A and GluN2B, respectively) in the rat hippocampus was determined by qPCR (Fig. 1a,b). Grin2b mRNA expression was maximal between P1 and P7 after which expression decreased gradually and was sustained at approximately half of peak levels (Fig. 1a) consistent with previous reports [4]. Conversely, Grin2a mRNA expression was very low at P1, increased after P7 and plateaued at P20 (Fig. 1b). TargetScan (www.targetscan.org) [19] version 6.2 and miRDB (www.mirdb.org) [20] were used to find candidate miRNAs that target NMDAR mRNAs. miRDB had one target for Grin2b mRNA (miR-539) and no targets for Grin2a. TargetScan had 12 targets for Grin2a and 68 targets for Grin2b mRNA, one of which was miR-539. We further narrowed the candidate list by choosing miRNAs that were selective for single NMDAR subunits and chose the candidates presenting the best seed matches and context scores (site-type, 3′-supplementary pairing, local AU content, position contribution as detailed in [21]) among this subset. This analysis produced three candidates for Grin2a (miR-19a, −351, and −137) and three candidates for Grin2b (miR-539, −3541, −296). We measured the expression levels of these candidate miRNAs in rat hippocampus by qPCR over the same developmental interval as Grin2a and Grin2b mRNAs. Only miR-19a and miR-539 showed an expression pattern that was inversely correlated with its predicted NMDAR subunit target (correlation coefficients: r = -0.8147 for Grin2a/miR-19a and r = -0.8134 for Grin2b/miR-539) (Fig. 1a,b). MiR-19a levels fell steeply shortly after birth and then flattened out by P11. In contrast, miR-539 showed a gradual increase beginning after birth and through the first month of post-natal life. Other candidate miRNAs (miR-351, miR-137, miR-3541 and miR-296) showed uncorrelated patterns of expression or did not vary with time (not shown).

**Fig. 2:**
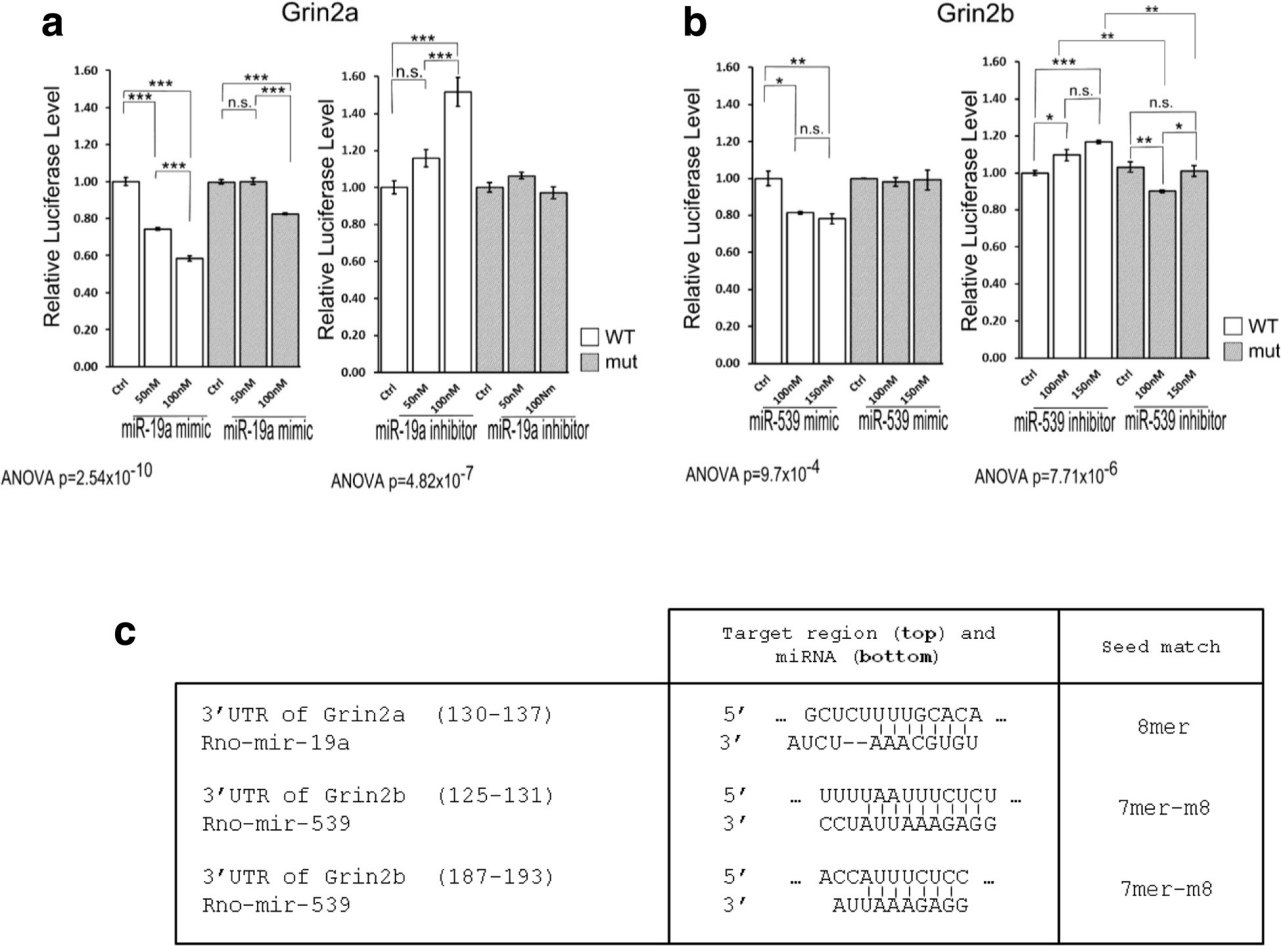
**a**. Validation of the Grin2a mRNA 3′UTR as a miR-19a target: relative Grin2a mRNA 3′UTR luciferase levels with 50 nM or 100 nM of miR-19a mimic measured 24 hrs after transfection (Mut., mutant UTR with a 3 bp mutation of the miR-19a target site; Ctrl, values obtained with the mirVana miRNA mimic Control) and relative Grin2a mRNA 3′UTR luciferase levels 24 h after transfection with an anti-miR-19a (concentrations of 50 nM and 100 nM) (Ctrl, transfection with a LNA Inhibitor Control). **b**. Validation of the Grin2b mRNA 3′UTR as a miR-539 target: relative Grin2b mRNA 3′UTR luciferase levels with 100 nM or 150 nM of miR-539 mimic 24 h after transfection (the concentrations used are higher than those for miR-19a because the Grin2b mRNA 3′UTR possesses two miR-539 target sites) (Mut., mutant UTR with a 3 bp mutation of the miR-539 target site; Ctrl, values obtained with the mirVana miRNA mimic Control) and relative Grin2b mRNA 3′UTR luciferase levels 24 h after transfection with an anti-miR-539 (concentrations of 100 nM and 150 nM) (Ctrl, transfection with a LNA Inhibitor Control). **c**. miR-19a and miR-539 binding sequences in the 3′UTR of Grin2a and Grin2b respectively, and the corresponding seed matches

To demonstrate that miR-19a and miR-539 target mRNAs encoding GluN2A and GluN2B respectively we performed luciferase assays (Fig. 2a,b). HEK293 cells co-transfected with a miR-19a mimic showed decreased luciferase activity of Luc-Grin2a 3′UTR by 41.6 % (±1.9 % of control, p < 0.001), whereas the mimic had much less effect on Luc-Grin2a-mut 3′UTR (−17.5 ± 0.5 %, p < 0.001). This finding is consistent with miR-19a binding to the 3′ UTR of Grin2a mRNA as a putative target. Co-transfection with the miR-19a locked nucleic acid (LNA) inhibitor increased the luciferase activity of Luc-Grin2a 3′UTR (51.7 ± 9.6 %, p < 0.001) with no significant effect on Luc-Grin2a-mut 3′UTR, confirming the target relationship (Fig. 2a). Similarly, co-transfection with the miR-539 mimic decreased the luciferase activity from Luc-Grin2b 3′UTR by 21.9 % (± 3.4 %, p < 0.01) but not from Luc-Grin2b-mut 3′UTR (1 %, p > 0.05) (Fig. 2b). Co-transfection with the miR-539 inhibitor increased the luciferase activity from Luc-Grin2b 3′UTR by 16.8 % (± 1.1 %, p < 0.001), whereas no significant change was observed for the Luc-Grin2b-mut 3′UTR between control and the maximal concentration (150nM) (p > 0.05) (Fig. 2b), confirming that miR-539 targets Grin2b mRNA. The concentration of miR-539 mimic required to attenuate luciferase activity in these experiments was higher than the concentration of miR-19a mimics, consistent with the presence of two miRNA response elements (MREs) for miR-539 in Grin2b mRNA (Fig. 2c).

**Fig. 3:**
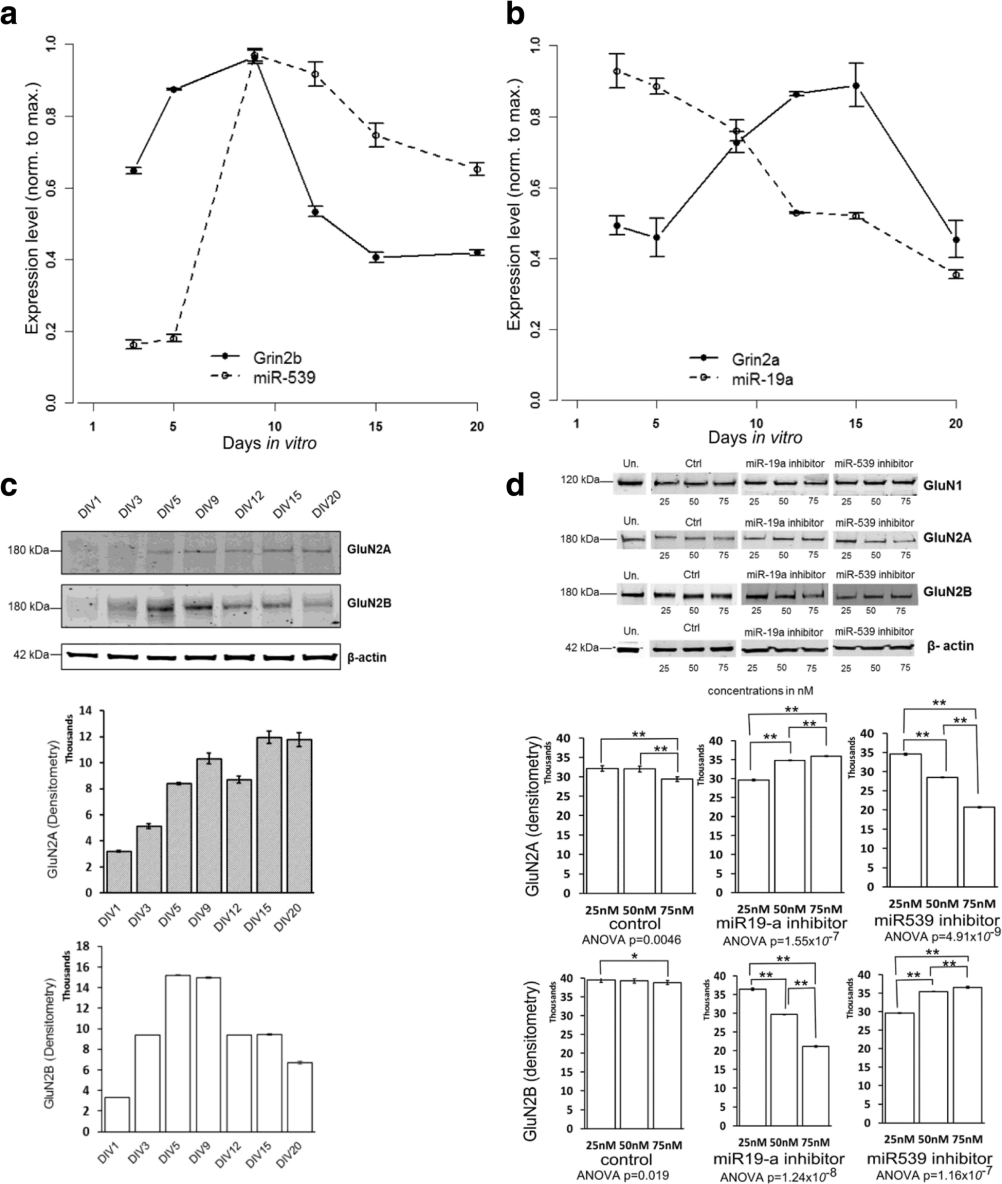
**a**, **b** *.* Relative mRNA levels obtained for Grin2a and Grin2b in rat hippocampal cultures*.* RNA was extracted between DIV3 (Day *In Vitro* 3) and DIV20. The values represented are normalized to GAPDH by SYBR qRT-PCR. The expression of their corresponding miRNAs (miR-19a and miR-539) are also represented and normalized to U6. **c**. Protein expression obtained for GluN2A, GluN2B and the loading control β-actin by Western-Blot. **d**. Expression of the GluN2A and GluN2B proteins after miRNA inhibitor treatment by Western blot. At DIV8, the rat hippocampal neurons were treated with 25 nM, 50 nM or 75 nM of miR-19a, miR-539 inhibitors, with a control (Ctrl) or untreated (Un.).Data in histograms were quantified with Image J software (n = 3). The values for the protein levels are normalized to β-actin

Cultured hippocampal neurons are a well characterized experimental system and are more easily manipulated than the intact hippocampus. To confirm that the developmental profile of NMDAR subunit expression from cultured neurons compared favorably with our results from acutely isolated hippocampal tissue, we extracted RNA from hippocampal cultures between DIV3 and DIV20. The time-dependent changes in the expression of Grin2a and Grin2b mRNA paralleled the changes observed in tissue (Fig. 3a,b). Western blots confirmed that GluN2A and GluN2B protein levels followed the mRNA expression pattern (Fig. 3c). MiR-19a and miR-539 approximated the expected inverse pattern of the targeted subunit: miR-19a expression gradually declined after DIV9, and miR-539 expression increased sharply. The initial increase in GluN2B protein (from DIV1 to DIV5) likely resulted from regrowth of dendrites following cell plating [22] and reduced the strength of the correlations.

To examine the role of these miRNAs in determining NMDAR subunit expression patterns, we used LNA miRNA inhibition. Based on the above observations, DIV8 was selected for the treatment. It was not possible to follow the expressions of miRNAs with this technique due to the recognized problem that the antisense inhibitor can directly inhibit the qPCR reaction [23], an issue also recognized when performing Northern blots [24]. Grin2a and Grin2b mRNA levels were not notably changed following LNA treatment (not shown). The absence of a miRNA effect on a validated target mRNA is frequently observed and is probably due to translational inhibition in the absence of mRNA target degradation. Therefore, the protein level was used to detect miRNA efficacy. We next examined whether miR-539 and miR-19a affect NMDAR subunit protein expression. We assessed GluN2A and GluN2B protein expression on cultured rat neurons following LNA treatment at DIV8 (Fig. 3d). In neurons treated with the miR-19a inhibitor, protein expression of GluN2A increased (21.3 ± 0.6 % of control, p < 0.01) and in neurons treated with the miR-539 inhibitor, protein expression of GluN2B increased (12.5 ± 0.1 % for GluN2B, p < 0.01). Interestingly, each of the miR inhibitors also induced a reciprocal effect in the regulated subunit that it does not directly target. The miR-19a inhibitor was associated with a decrease in GluN2B decreased (−29.5 ± 0.71 %, p < 0.01) and miR-539 inhibitor was associated with a decrease in GluN2A (−40 ± 0.52 % of control, p < 0.01). These findings support the roles of miR-19a and miR-539 on their targets and suggest additional controls that maintain reciprocal levels of the mature and immature subunits. To examine whether other NMDA receptor subunits were affected by LNA treatment, we used an antibody for GluN1 but detected no difference in protein levels following LNA treatment (Fig. 3d).

### The GluN2 subunit switching network is linked to REST

**Fig. 4:**
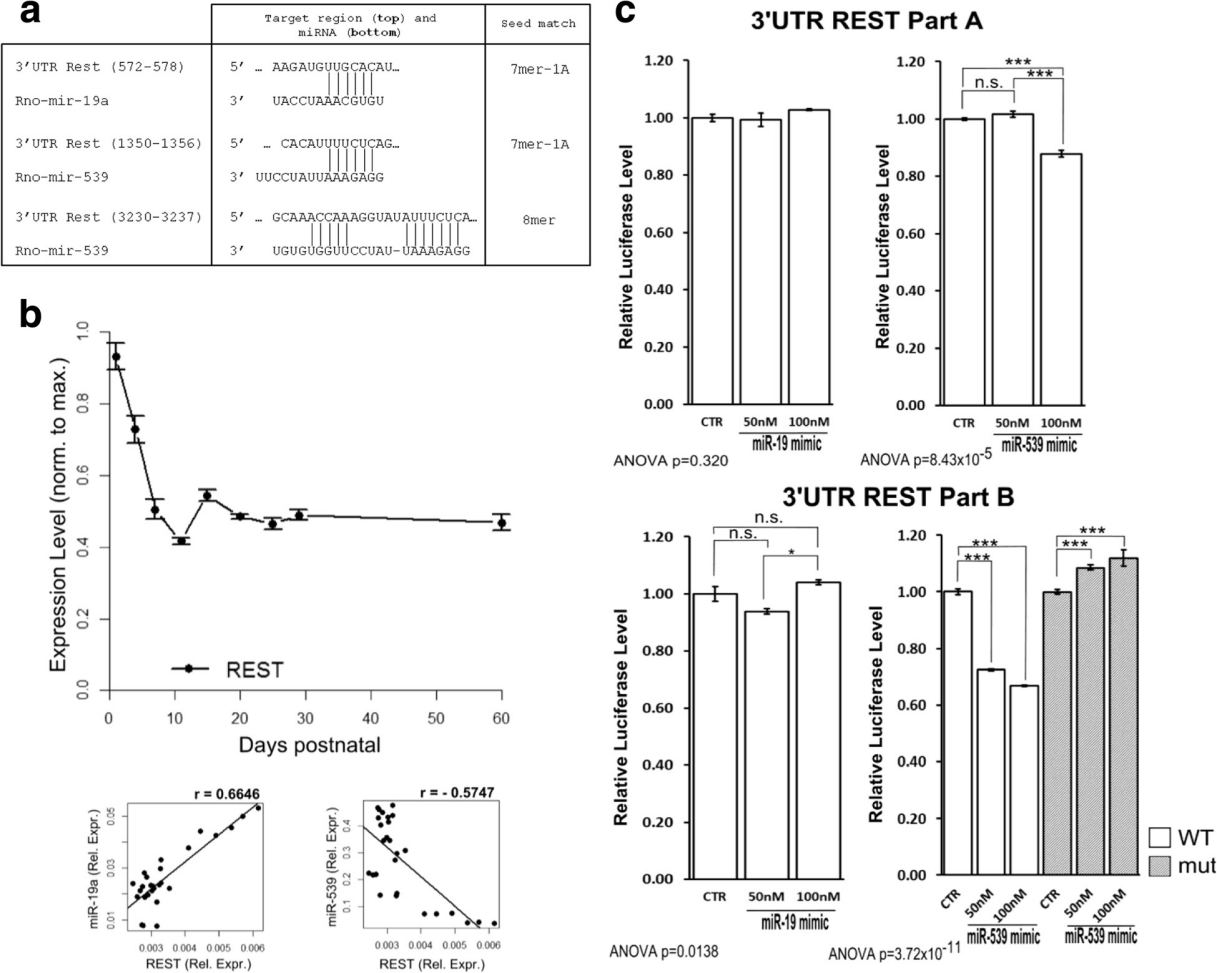
The role of REST in miRNA regulation of NMDAR subunit expression. **a**. Putative miR-19a and miR-539 binding sequences in the 3′UTR of REST*.* **b**. qPCR analysis of REST mRNA expression. Its expression markedly decreased from P1 to P11 followed by a plateau into the adult (P60). REST negatively correlates with miR-539 (The Pearson’s correlation coefficient is r = -0.5747 for REST/miR-539). **c**. *PartA* of the REST 3′ UTR contains a MRE for miR-19a and *PartB* contains two MREs for miR-539. Only the miR-539 mimic effectively targeted the 3′UTR of REST containing the MREs for miR-539. A weak increase was observed on the Luc-REST*-*mut 3′UTR with the miR-539 mimic (n = 3) (mut.: 3′UTR mutated)

The repressor element 1 silencing transcription factor (REST) silences genes by epigenetic remodeling [15]. Grin2b is a REST target and knockdown of REST *in vivo* prevents the developmental decline in GluN2B, but not GluN2A. Interestingly, the 3′ UTR of REST has putative sites for miR-19a and miR-539 (Fig. 4a). To relate the REST expression pattern to those of the GluN2 subunits, miR-19a and miR-539 expressions, we tracked REST mRNA expression *in vitro* by qPCR (Fig. 4b). REST expression dramatically decreased from P1 to P11, then plateaued through adulthood (P > 60). To test whether REST was a target for miR-19a and/or miR-539 we used the luciferase assay. The miR-539 mimic reduced the luciferase signal when fused to the 3′ UTR of REST and especially for the REST PartB (−33.12 ± 0.39 % of the signal compared to the control, p < 0.001). On the other hand, the miR-19a mimic had no effect on the luciferase signal (3.92 ± 1.06 % of the signal compared to the control (REST PartB, 50nM), p < 0.05) (Fig. 4c). Thus, it is likely that REST operates within the gene regulatory network in conjunction with the implicated miRNAs to regulate NMDAR maturation.

## Discussion

**Fig. 5:**
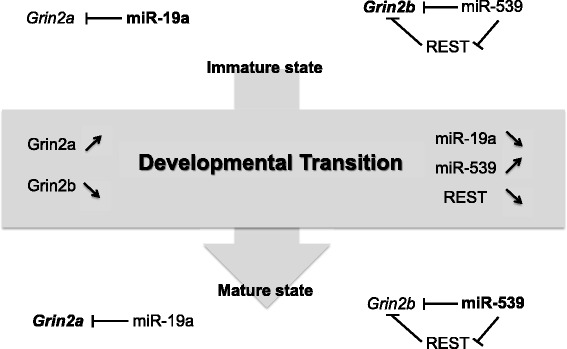
Summary circuitry. The two states (immature/mature) are represented and the developmental transition also. Some RNAs or miRNAs are written in bold to precise their highest expression. Our data show that Grin2a expression is controlled by miR-19a whereas Grin2b is included in a feed forward inhibitory motif

The regulation of changes in NMDAR subunit composition during development suggests a complex graded dynamical system: as the GluN2 subunit composition changes during maturation, miR-539 goes up and REST goes down (Fig. 5). Concomitantly, miR-19a decreases as GluN2A reaches its mature level. REST targeting by miR-539 may smoothly implement the transition by directly or indirectly coordinating the developmental reduction of both Grin2b and REST*.* These transitions require an extensively parameterized network of control elements capable of operating as a closed-loop system from the time of induction until a new equilibrium is reached. miRNAs act in small-scale gene regulatory networks with defined topologies [25, 26]. These network motifs function in recurrent regulatory circuits, often with transcription factors [27] and, in this case, implement transitions in the NMDAR subunit composition.

## Methods

### Ethics approval

All animal care procedures were reviewed and approved by the Institutional Animal Care Committee at the University of California, Santa Barbara and found to be in compliance with guidelines on Animal Care.

### Hippocampus rat brain extraction and rat primary hippocampal neuron cultures

Hippocampi from postnatal days (from P1 to P > 60) rats (strain Sprague-Dawley from Charles River Laboratories) were dissected. Neurons for culture were dissected from E18 Sprague Dawley rats in 5 mL of dissection media containing 500 μL of Trypsin 2.5 % and grown in complete growth medium (500 mL Neurobasal media, 10 mL B-27 Supplement, 1.25 mL L-Glutamine and 1 mL P/S).

### RNA extraction

Total RNA containing microRNA was extracted from the frozen hippocampi of each animal or cultures by using a mirVana miRNA isolation kit (#AM1560, Life technologies).

### Western blot

After neuronal cells lysates obtainment, the following antibodies were used: anti-GluN2A (#AB1555P, Millipore); anti-GluN2B (#05-920, Millipore); anti-β-Actin (#A5441, Sigma) and anti-NMDAR1 (GluN1) (#MAB363, Chemicon International). Western Blots were quantitated using the Image J software.

### LNA treatment

At DIV8, neurons were transfected with either complementary sequence based locked nucleic acid inhibitors of miR-19a or miR-539 (miR-19a, #410118-00, Exiqon; miR-539, #MC11336, Exiqon), or a scrambled control (miRCURY LNA Inhibitor Control, #199004-00, Exiqon) using Lipofectamine 2000. The following day, protein lysates were collected.

### qPCR measurements

Quantitative PCRs were carried out using specific primers (Table 1) and SYBR Green PCR Master Mix (#4367659, Life Technologies). Grin2a, Grin2b and REST expressions were normalized to GAPDH expression. For miRNAs, 10 nanograms of the total RNA containing microRNA were reverse-transcribed with a TaqMan MicroRNA Reverse Transcriptase kit (#PN 4366596, Applied Biosystems) and analyzed with the TaqMan MicroRNA Assay containing specific primers for miR-19a or miR-539 (hsa-miR-19a-3p, #000395 and hsa-miR-539-5p, #001286, Applied Biosystems) and U6 as a control (#001973).

**Table 1:** Sequences of primers for mRNA detection

Gene symbol	Primer (5′…3′)
Grin2a(GluN2A)-F	gggctgctcttctccatcagc
Grin2a(GluN2A)-R	cccttgtctgaaaccatgtccac
Grin2b(GluN2B)-F	tggccctcagcctcatcacc
Grin2b(GluN2B)-R	catcacggattggcgctcct
Gapdh-F	gggcatcctgggctacactga
Gapdh-R	ccttgctgggctgggtggt

### Luciferase assay

The 3′UTR of the rat Grin2a or Grin2b mRNA were generated by PCR and inserted between the SpeI and HindIII restriction sites downstream of the Firefly Luciferase of the plasmid pMIR-REPORT system (#AM5795, Applied Biosystems). Due to the size of the rat 3′UTR of REST, its sequence was inserted between the SacI and PmeI restriction sites in two different parts: PartA (1217 bp), which contains the MRE for miR-19a and PartB (2176 bp) which contains the two MREs for miR-539.

The 3-nucleotides mutants of the Luc-3′UTR Grin2a, Luc-3′UTR Grin2b, Luc-3′UTR REST Part A and Part B were prepared using the primers indicated in (Table 2).

**Table 2:** Primers for mutagenesis

Mutated sequence	Primer (5′…3′)
FLuc-Grin2a-mut 3′UTR-F	gggaggtactttttgttggctcttttaagcatggctccatgccataat
FLuc-Grin2a-mut 3′UTR-R	attatggcatggagccatgcttaaaagagccaacaaaaagtacctccc
FLuc-Grin2b-mut1 3′UTR-F	tggctcccattcgctttttcttcttcttttaatgcttctatgggatcctggag
FLuc-Grin2b-mut1 3′UTR-R	ctccaggatcccatagaagcattaaaagaagaagaaaaagcgaatgggagcca
FLuc-Grin2b-mut2 3′UTR-F	aaccctcgtggccagcaccatgcttcctccctcgc
FLuc-Grin2b-mut2 3′UTR-R	gcgagggaggaagcatggtgctggccacgagggtt
FLuc-Rest-mut 3′UTR PartA-F	ctttggtgcttttctggcttaagatgttaagcatggttcttgtttttgtttctttaacc
FLuc-Rest-mut 3′UTR PartA-R	ggttaaagaaacaaaaacaagaaccatgcttaacatcttaagccagaaaagcaccaaag
FLuc-Rest-mut1 3′UTR PartB-F	taactaagtacagtccggattttaagtatcacattgcttcagggatctccacaaac
FLuc-Rest-mut1 3′UTR PartB-R	gtttgtggagatccctgaagcaatgtgatacttaaaatccggactgtacttagtta
FLuc-Rest-mut2 3′UTR PartB-F	gttactttcctgaggcaaaccaaaggtatatgcttcaaggttctgctgcc
FLuc-Rest-mut2 3′UTR PartB-R	ggcagcagaaccttgaagcatatacctttggtttgcctcaggaaagtaac

HEK293T cells were transfected with phRL-TK, the FLuc-3′UTR Grin2a or FLuc-3′UTR Grin2b or FLuc-3′UTR REST and the corresponding miRNA mimics (miRVana miR-19a mimic, #MC10649, Ambion; miRVana miR-539 mimic, #MC11336, Ambion and a miRVana miRNA mimic Negative Control, #4464058, Life Technologies) or miRNA inhibitors (miR-19a, #410118-00, Exiqon; miR-539, #410328-00, Exiqon or miRCURY LNA Inhibitor Control, #199004-00, Exiqon).

### Statistical analysis

Data were expressed as mean ± SEM. One-way ANOVA followed by Tukey’s post-hoc comparisons tests were performed in all statistical. Significance levels are *: *p* < 0.05; **: *p* < 0.01; ***: *p* < 0.001; n.s.: not significant and r: Pearson’s correlation coefficient.

## Abbreviations

DIV: Days in vitro
NMDAR: N-Methyl-D-Aspartate Receptor
miRNA: microRNA
MRE: miRNA Response Element
LTP: Long-Term Potentiation
LNA: Locked Nucleic Acid
